# Supplementary material for: DMRforPairs: identifying Differentially Methylated Regions between unique samples using array based methylation profiles
Source: BMC Bioinformatics. 2014 May 15;15:141. doi: 10.1186/1471-2105-15-141 (PMC4046028; doi:10.1186/1471-2105-15-141)
Supplement: Additional file 2 — DMRforPairs output for the comparison of A431-MCF7 and NA17018-NA17105. Please start from the HTML files in each folder. Available via the BMC Bioinformatics website. [file 1471-2105-15-141-S2.zip › 1394847754114233_MOESM2_ESM/A431_MCF7/figures/34910.pdf]

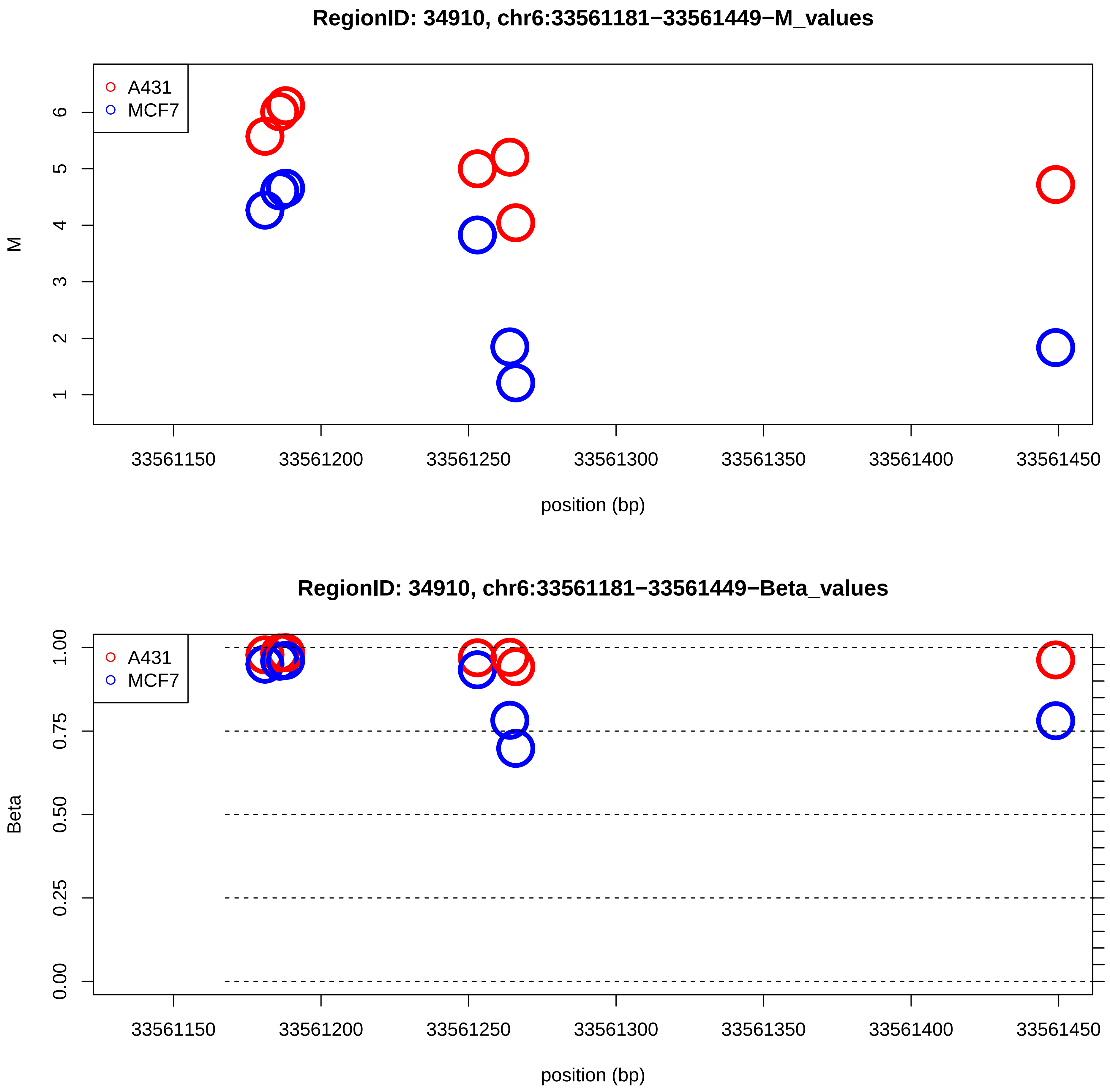
RegionID: 34910, chr6:33561181−33561449−M_values
A431
MCF7
M
1
2
3
4
5
6
33561150
33561200
33561250
33561300
33561350
33561400
33561450
position (bp)
RegionID: 34910, chr6:33561181−33561449−Beta_values
A431
MCF7
Beta
0.00
0.25
0.50
0.75
1.00
33561150
33561200
33561250
33561300
33561350
33561400
33561450
position (bp)

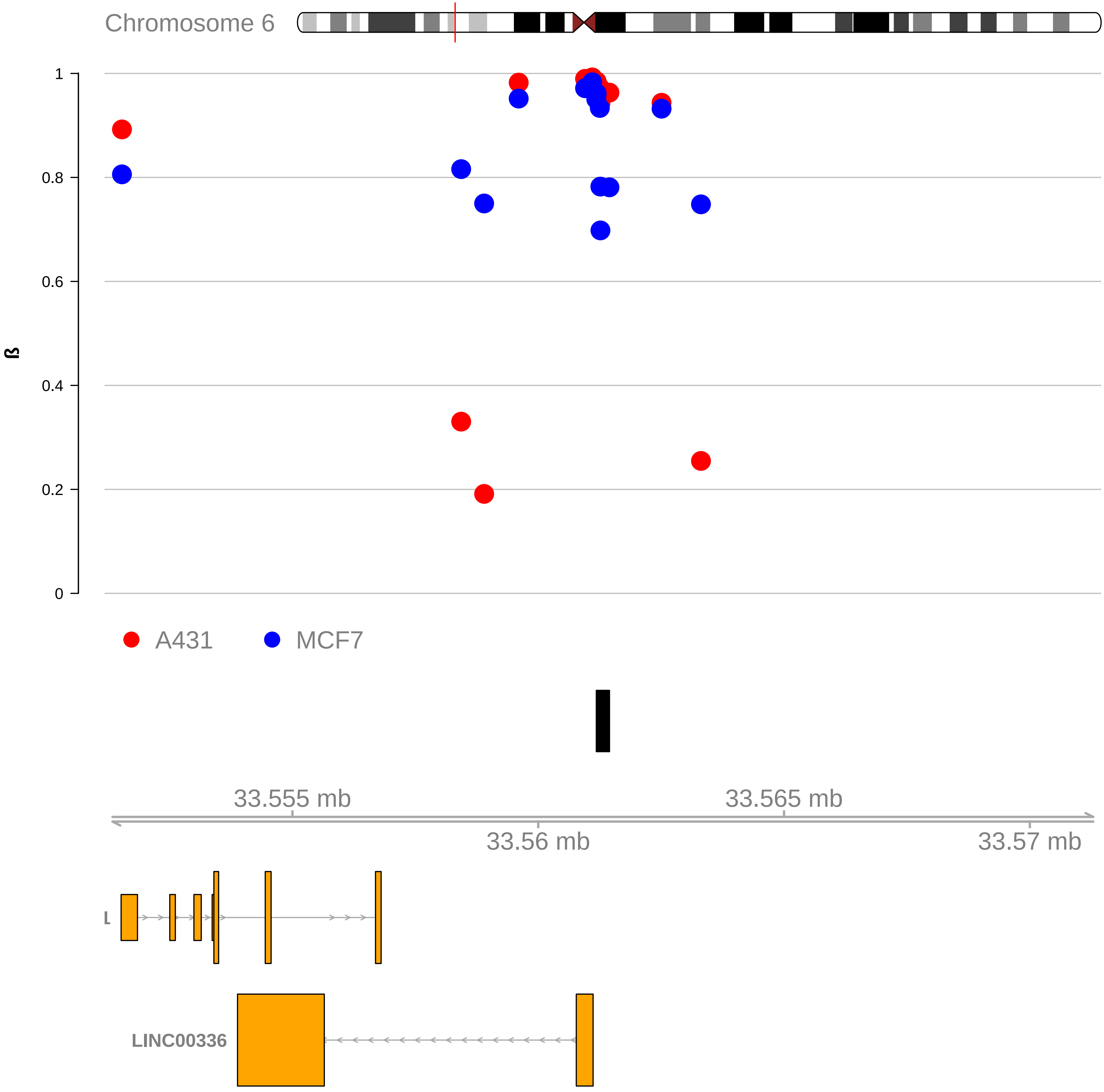

Chromosome 6
1
0.8
0.6
0.4
0.2
0
ß
A431
MCF7
33.555 mb
33.565 mb
33.56 mb
33.57 mb
LINC00336
